# Polyethylene Glycol Misuse Causing Acute Renal Failure and Metabolic Acidosis Requiring Dialysis: A Case Report

**DOI:** 10.7759/cureus.65838

**Published:** 2024-07-31

**Authors:** Timothy J Kolosionek, Rena Y Jiang, Mostafa M Meleis, Natalie E Ebeling-Koning, Ryan M Surmaitis

**Affiliations:** 1 Department of Emergency and Hospital Medicine, Lehigh Valley Health Network/University of South Florida Morsani College of Medicine, Allentown, USA; 2 Division of Medical Toxicology, Department of Emergency and Hospital Medicine, Lehigh Valley Health Network/University of South Florida Morsani College of Medicine, Allentown, USA

**Keywords:** polyethylene glycol, laxative misuse, acute renal failure, anion-gap metabolic acidosis, osmotic laxative

## Abstract

Laxative misuse is a well-known occurrence, most often identified in patients struggling with eating disorders. Polyethylene glycol (PEG) 3350 is a readily available, well-tolerated osmotic laxative. High doses of PEG 3350 may cause gastrointestinal upset, diarrhea, dehydration, and electrolyte imbalance, although systemic toxicity is infrequently reported. This case report highlights the exceedingly rare metabolic derangements associated with profound levels of protracted PEG misuse. A 60-year-old female presented to the emergency department with altered mental status. She was found to have acute renal failure (ARF), anion gap metabolic acidosis (AGMA), and rhabdomyolysis secondary to excessive PEG 3350 use, requiring continuous renal replacement therapy (CRRT). Renal function improved after three days of CRRT, and no alternative causes beyond PEG ingestion were found to account for her mental status changes or metabolic anomalies. This report illustrates the importance of considering osmotic laxative misuse in the setting of pre-renal and intrinsic renal failure.

## Introduction

Laxative misuse is very commonly observed, especially among individuals struggling with eating disorders [1-3]. In 2021, 15,263 laxative-related calls were made to American poison centers; the majority of cases involved children, and only 1,099 cases were evaluated in a hospital [4]. Of the numerous classes of laxatives, osmotic laxatives are less commonly misused, potentially due to the large volume of fluid required for each dose [1].

Polyethylene glycol (PEG) 3350 is a readily available, well-tolerated osmotic laxative [5]. Osmotic laxatives facilitate the passage of stool by increasing osmotic pressure in the gut lumen [1]. They are generally associated with stable hydration and electrolyte status. Side effects of PEG 3350 include flatulence, nausea, abdominal cramps, diarrhea, abdominal distension, and rectal hemorrhage [6]. However, the incidence of severe adverse effects in adults is low [6]. PEG 3350 specifically seems a safe and effective choice for longer-term use in elderly people [7,8], a population with prevalent laxative use due to a high incidence of constipation [1]. Multiple laxative classes, including bulk-forming, osmotic, and stimulant agents, have been safely used in elderly patients for periods of less than three months [7].

Given the relative safety of over-the-counter laxatives, especially PEG, it follows that only 162 of the above 15,263 laxative exposures were associated with non-minor outcomes [4]. We report a case that demonstrates the exceedingly rare metabolic derangements associated with profound levels of protracted PEG misuse, including acute renal failure (ARF), anion gap metabolic acidosis (AGMA), and rhabdomyolysis requiring continuous renal replacement therapy (CRRT).

This case report was presented in part as an abstract at the American College of Medical Toxicology Annual Meeting (April 12, 2024, Washington, DC), and the Pennsylvania College of Emergency Physicians Scientific Assembly (May 2, 2024, Pocono Manor, PA).

## Case presentation

A 60-year-old female with a history of stage three chronic kidney disease, hypertension, and depression presented to the emergency department with altered mental status. The patient could not recount the circumstances that had led to her presentation. She reported dizziness and admitted that she had fallen. She denied any recent illness, fever, dyspnea, pain, or nausea. She voiced concerns about constipation and admitted to ingesting at least “one bottle” of PEG 3350 daily for at least 30 days. At the time of presentation, she could not reliably comment on other medication use or ingestions. Her boyfriend stated that she had become increasingly fatigued over the preceding two days. On the day of the presentation, he had found the patient confused on the bathroom floor, having last seen her at least two hours prior. He also stated the patient had been having at least 20 bowel movements per day.

Her medication list in the electronic medical record indicated that she was taking calcium carbonate, cholecalciferol, estrogen-medroxyprogesterone, fluticasone, hydroxyzine, levothyroxine linaclotide, lisinopril, melatonin, mirtazapine, oxybutynin, pantoprazole, polyethylene glycol, quetiapine, rosuvastatin, albuterol (inhaler), and cariprazine. Her boyfriend noted that of these medications, two psychiatric medications were new (cariprazine and mirtazapine). The patient had no known history of an eating disorder. On evaluation, the patient was awake but not oriented, was notably fatigued, and could only answer simple questions. Initial vital signs included a blood pressure of 120/54 mmHg, heart rate of 96 beats per minute, temperature of 97.4 °F (36.3 °C), respiration rate of 14 breaths per minute, and oxygen saturation of 96%. Notable physical exam findings included very dry mucous membranes and disorientation.

**Figure 1:**
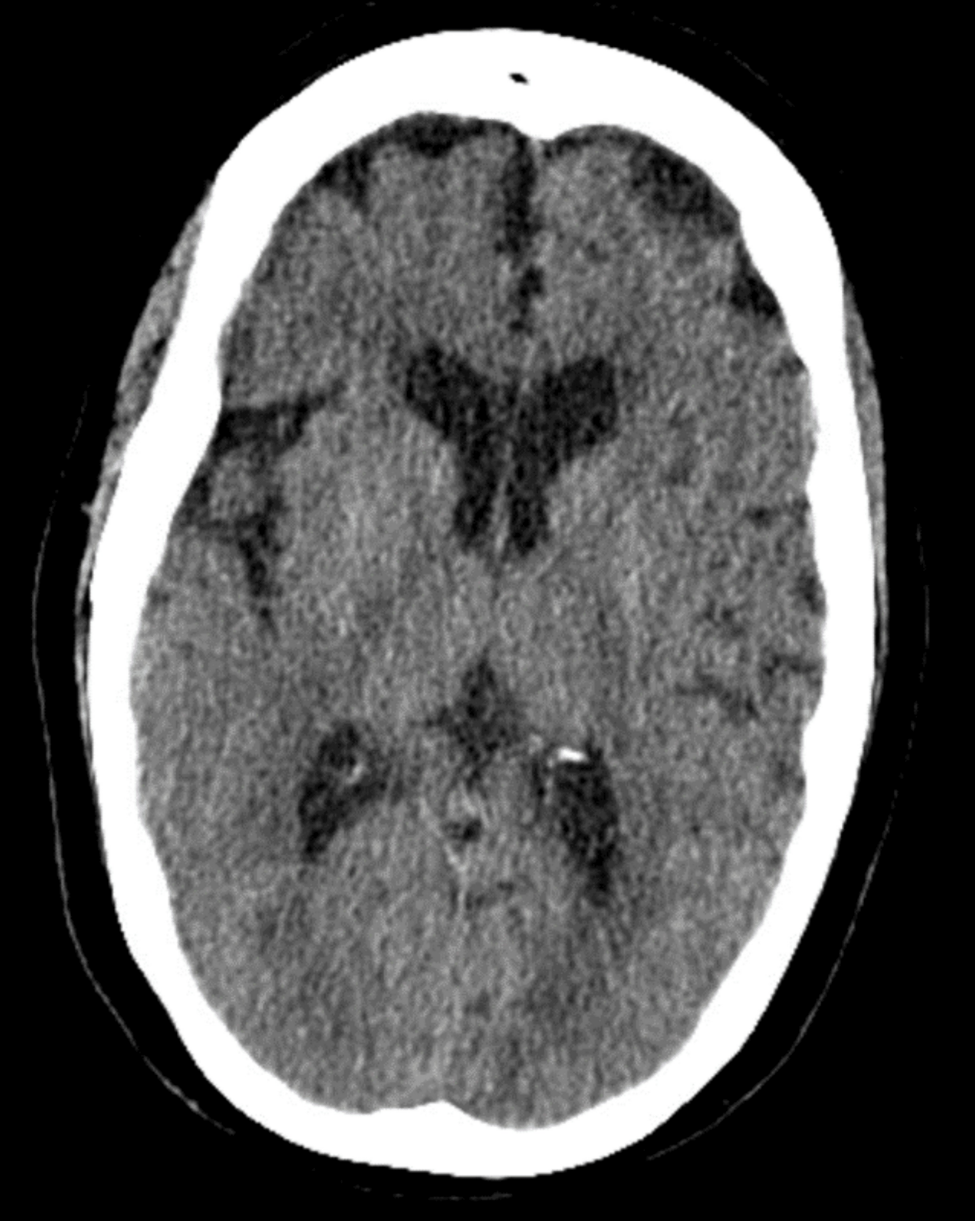
Representative axial section of a normal CT scan of the head CT: computed tomography

**Figure 2:**
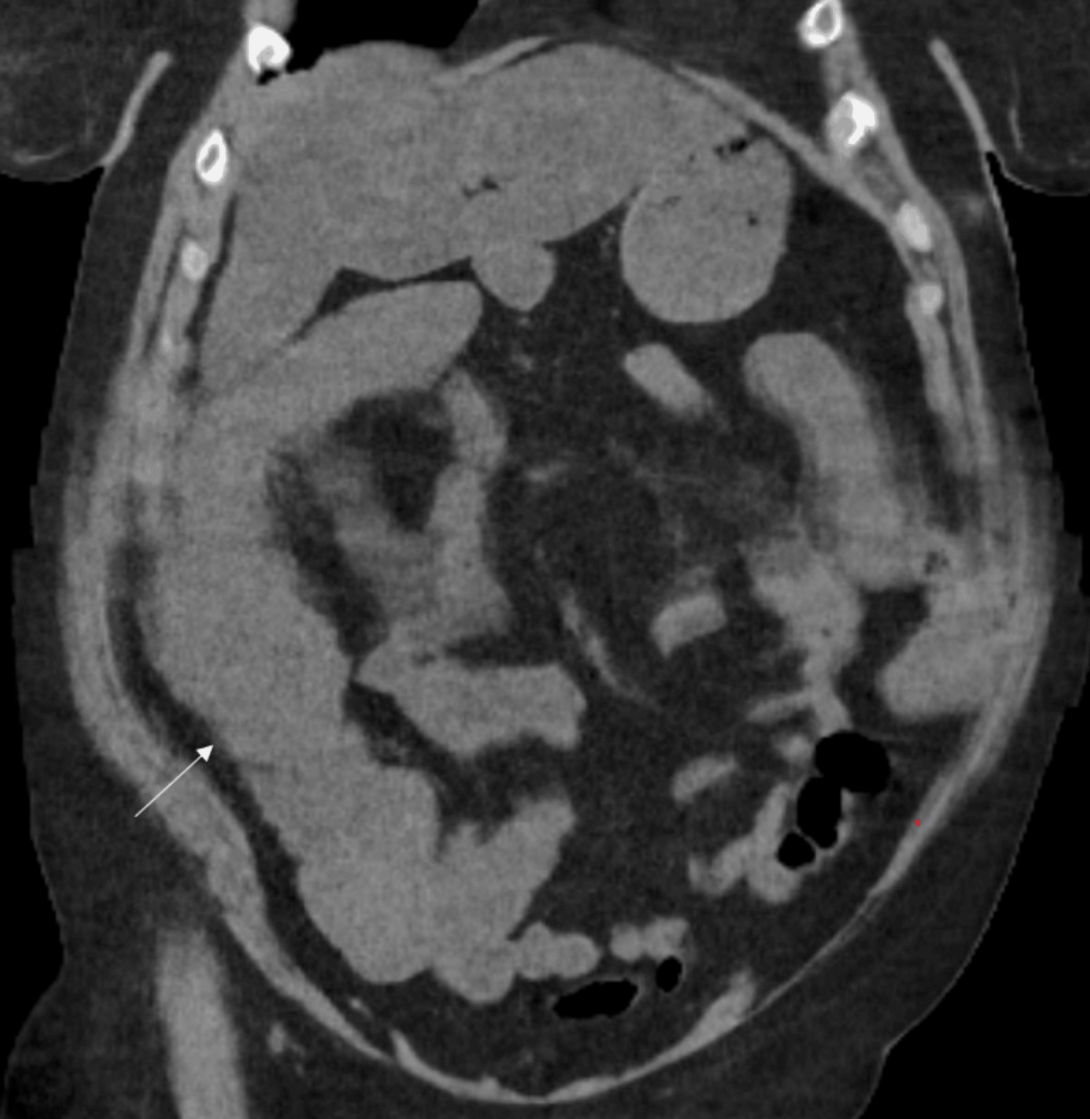
Representative coronal section of an abdominal CT scan showing fluid-filled large bowel loops (white arrow) CT: computed tomography

Laboratory findings (Table 1) were significant for ARF, AGMA, rhabdomyolysis, and elevated lactate. CT of the head was negative (Figure 1). CT of the chest, abdomen, and pelvis without contrast showed fluid-filled large bowel loops (Figure 2). Given her significantly high AGMA without reliable history, the patient was empirically started on fomepizole. Bicarbonate infusion was initiated, and the patient was admitted to the ICU for emergent dialysis.

**Table 1:** Notable initial laboratory values BUN: blood urea nitrogen; CK: creatine kinase

Test	Result	Reference range	Units
BUN	180	7-25	mg/dL
Creatinine	21.51	0.40-1.10	mg/dL
Anion gap	37	3-11	N/A
Bicarbonate	5	21-31	mmol/L
pH	7.07	7.31-7.41	N/A
pCO_2_	<19	41-51	mmHg
HCO_3_^-^	5	23-29	mEq/L
CK	11,493	30-223	U/L
Lactate	3.3	0.5-2.2	mmol/L

The patient underwent three days of CRRT, complicated by hypotension requiring vasopressor infusion. She developed hyperphosphatemia, likely due to rhabdomyolysis. Acetaminophen, salicylate, toxic alcohol panel, and urine drug screen were negative. Her mental status and laboratory derangements improved dramatically over the three days: BUN: 29 mg/dL, creatinine: 2.59 mg/dL, anion gap: 4, HCO_3_: 27 mEq/L. She explained that she often felt constipated and thus consumed 1-1.5 bottles of PEG 3350 (estimated to be 765 grams based on the described bottle size) per day, yet had numerous episodes of diarrhea during the hospital course. No further workup of the diarrhea was performed as part of the hospital stay. Nephrology, medical toxicology, neurology, and psychiatry were consulted, and found no alternative causes beyond PEG ingestion for her mental status changes or metabolic anomalies. Renal function continued to normalize after the cessation of CRRT, and the patient was discharged on hospital day 10 to an inpatient physical rehabilitation facility.

## Discussion

Systemic toxicity due to PEG 3350 is rarely reported, likely because only 0.06% of it is absorbed from the gut lumen into systemic circulation in people with normal gut mucosa [9,10]. Chronic excessive PEG 3350 use, however, may lead to higher circulating levels than anticipated with normal doses. Our patient’s estimated PEG 3350 exposure - one 765 g bottle daily for at least 30 days - was 45 times higher than the recommended daily dose of 17 g for a maximum of seven days [11]. Although PEG 3350 is not intended to be used for more than seven consecutive days, an open-label study demonstrated the safe and effective use of PEG 3350 for treating chronic constipation over 12 months [8]. No tachyphylaxis or clinically significant changes in electrolytes were observed at the recommended dose [8].

PEG laxatives are thought to be metabolically inactive when ingested [5] and maintain both euvolemia and stable electrolyte levels at therapeutic dosing [1]. Animal studies have failed to demonstrate metabolic changes when PEG comprised up to 2% of the daily diet over one year [5]. Furthermore, human pharmacokinetic data illustrate that PEG is almost exclusively excreted unchanged in the stool [9]. Consequently, ill effects from PEG ingestion, such as dehydration and electrolyte imbalance [1], are likely the result of electrolyte changes induced by subsequent stool output. Fortunately, these effects are usually self-limiting and not life-threatening [1].

PEG has been previously associated with AGMA and ARF, but only in lower molecular weight formulations [12-14]. Two case reports involving patients receiving high doses of intravenous (IV) lorazepam have been published [12,13]. It is theorized that the PEG 400 diluent is oxidized to hydroxy acid and diacid metabolites via alcohol dehydrogenase [13]. However, absorption of enteral PEG is minimal, as above [9], therefore making this mechanism less likely in this patient’s condition. No other reports of chronic over-ingestion of PEG, as seen in this patient, were identified in the literature.

The immense volume of chronic PEG ingested by this patient led to a highly elevated AGMA with secondary metabolic alkalosis, unlike the primary metabolic alkalosis associated with laxative misuse [15]. Although the patient may have had an isolated metabolic alkalosis early in her laxative use course, the volume loss prompted by her overuse of an osmotic laxative likely induced her severe renal failure and azotemia. This was undoubtedly confounded by the patient’s preexisting stage three kidney disease. Physical exam features suggested a hypovolemic state, including dry mucus membranes and hypotension initially responsive to IV fluids. The BUN:creatinine ratio on arrival was suggestive of an intrinsic-renal process. Her hypovolemic hypotensive state presumably caused a pre-renal kidney injury, but ultimately led to acute tubular necrosis as the hypotensive state persisted.

The marked azotemia presumably led to the anion gap, as other toxicologic causes, such as ethanol, methanol, ethylene glycol, glycolic acid, acetaminophen, and salicylate levels, were undetectable. Furthermore, the slightly elevated lactate was insufficient to explain the observed anion gap. Regardless, renal replacement therapy would have addressed many of these causes of an elevated anion gap. Fortunately, renal function recovered following volume resuscitation and temporary dialysis.

## Conclusions

To our knowledge, this is one of the first reported cases of AGMA, ARF, and rhabdomyolysis due to chronic excessive PEG 3350 exposure necessitating CRRT. Our patient’s extreme use of PEG over a prolonged period highlights that drug data calculated at therapeutic dosing cannot be extrapolated to profound supratherapeutic dosing, regardless of intent. Moreover, products sold over the counter, including laxatives, cannot be assumed to be safe when patients deviate from the dosage recommended by medical professionals. Accordingly, the report demonstrates the importance of counseling patients on appropriate medication dosing, even for over-the-counter products, such as laxatives.
